# Towards a Semantic Web of Things: A Hybrid Semantic Annotation, Extraction, and Reasoning Framework for Cyber-Physical System

**DOI:** 10.3390/s17020403

**Published:** 2017-02-20

**Authors:** Zhenyu Wu, Yuan Xu, Yunong Yang, Chunhong Zhang, Xinning Zhu, Yang Ji

**Affiliations:** 1 Information Network Engineering Research Center of Ministry of Education, Beijing University of Posts and Telecommunications, Beijing 100876, China; ji.yang.0001@gmail.com; 2 State Key Laboratory of Networking and Switching Technology, Beijing University of Posts and Telecommunications, Beijing 100876, China; xyuanu2011@gmail.com (Y.X.); yangyunong@bupt.edu.cn (Y.Y.); zhangch@bupt.edu.cn (C.Z.); zhuxn@bupt.edu.cn (X.Z.)

**Keywords:** Web of Things, cyber-physical system, semantic web, semantic sensor network, ontology, entity linking, semantic annotation, semantic reasoning

## Abstract

Web of Things (WoT) facilitates the discovery and interoperability of Internet of Things (IoT) devices in a cyber-physical system (CPS). Moreover, a uniform knowledge representation of physical resources is quite necessary for further composition, collaboration, and decision-making process in CPS. Though several efforts have integrated semantics with WoT, such as knowledge engineering methods based on semantic sensor networks (SSN), it still could not represent the complex relationships between devices when dynamic composition and collaboration occur, and it totally depends on manual construction of a knowledge base with low scalability. In this paper, to addresses these limitations, we propose the semantic Web of Things (SWoT) framework for CPS (SWoT4CPS). SWoT4CPS provides a hybrid solution with both ontological engineering methods by extending SSN and machine learning methods based on an entity linking (EL) model. To testify to the feasibility and performance, we demonstrate the framework by implementing a temperature anomaly diagnosis and automatic control use case in a building automation system. Evaluation results on the EL method show that linking domain knowledge to DBpedia has a relative high accuracy and the time complexity is at a tolerant level. Advantages and disadvantages of SWoT4CPS with future work are also discussed.

## 1. Introduction

Web of Things (WoT) [1] aims at reusing the REpresentational State Transfer (REST) architectural style [2] and Web protocols to make networked physical objects first-class citizens of the World Wide Web. In the context of WoT, physical things are abstracted as building blocks of Web applications with uniform resource identifiers (URI), standard HyperText Transfer Protocol (HTTP) interfaces, and hypermedia-based representations. To enable discovery and interoperability of the Internet of Things (IoT) service on a worldwide basis, the W3C WoT Interest Group has proposed a distributed “Web Servient” [3] as a soft-defined virtualization middleware for physical things. In cyber-physical system (CPS) applications, the virtualization of devices with RESTful Web APIs lowers the barriers of developing IoT application across domains, especially in complex building automation, industry 4.0, or smart city applications. However, designing more uniform knowledge representations of devices is quite necessary to improve system interoperability and facilitate resource discovery, composition, and decision-making process in CPS applications. Accordingly, the Semantic Web of Things (SWoT) [4] is proposed, bridging semantic technology to the Web of Things, facilitating the creation of a networked knowledge infrastructure [5] with more interoperable meaningful data from both the physical and cyber world. Hence, high-level knowledge could be queried and inferred from details of the sensory observations to gain more contextual human-machine and machine-machine interactions.

There have been already several efforts to integrate semantic web with web-based IoT platforms including SENSEI [6], semantic sensor web [7], SPITFIRE [8], as well as work by the Kno.e.sis Center [9], CSIRO [10], and the Spanish Meteorological Agency [11]. By annotating sensor-related features, such as the network, deployment, data formats, etc., with machine-understandable vocabulary, i.e., semantic sensor network (SSN) ontology [12], it becomes possible to automate further tasks, e.g., deployment, maintenance, and integration of devices. Various studies ([13,14,15,16,17]) propose to extend and reuse existing knowledge-based representations to annotate metadata and stream sensory data for improving system interoperability and intelligent decision-support services based on semantic technologies. However, these efforts have limitations: (1) things are modeled using domain-specific vocabularies, which mainly consider deployment and maintenance use cases of sensor networks, but not CPS applications with collaborative and causal relationships among sensors and actuators, such as anomaly detection and diagnosis from sensor observations with automatic feedback via actuator control; (2) current domain knowledge is incompatible with the growing body of semantic global knowledge bases (KB)/Linked Open Data (LOD). This knowledge is fragmented in different applications, which lacks alignments and interlinking among concepts and facts, especially some global common sense knowledge, which will be very useful for knowledge acquisition and reasoning.

In this paper, we propose the SWoT for CPS (SWoT4CPS) framework, and it addresses these limitations by providing: (1) uniform SWoT-O vocabularies to integrate descriptive metadata of WoT resources and describe internal machine-to-machine collaborative rules by extending the SSN upper ontology; (2) an entity-linking (EL)-based methodology to annotate and extract semantics from domain-specific representations of WoT resources by linking domain knowledge to common sense KB; and (3) semantic search and rule-based semantic reasoning demonstrations of building automation applications to illustrate the WoT resource integration, anomaly diagnosis, and automatic control based on the hybrid domain and common sense KB.

The remainder of this paper is structured as follows: In Section 2, the previous work related to semantic sensor webs, KB construction, and related applications are summed up. Section 3 states the problem and challenges, as well as requirements of designing the SWoT4CPS framework. Section 4 mainly presents the architecture of the SWoT4CPS system, along with the details of the SWoT-O ontology and EL annotation models and algorithms. In Section 5, we propose a reference implementation and evaluate the EL-based annotation model to testify to the feasibility and performance of the SWoT4CPS framework. Section 6 concludes the paper with a summary and outline of the remaining steps, toward future work.

## 2. Related Work

In this section we provide an overview of related work on leveraging semantic technology to annotate sensor data. We summarize the methods into two main categories: (1) top-down methodology based on linked data and ontological knowledge engineering; and (2) bottom-up methodology based on machine learning models by extracting knowledge from existing data sources.

### 2.1. Semantic Sensor Web and Linked Sensor Data

There are some notable works that attempt to build semantic models and link semantic annotations for IoT. The semantic sensor web (SSW) proposes annotating sensor data with spatial, temporal, and thematic semantic metadata [6]. This approach uses the current Open Geospatial Consortium (OGC) and Sensor Web Enablement (SWE) specifications and attempts to extend them with semantic web technologies to provide enhanced descriptions to facilitate access to sensor data. The W3C Semantic Sensor Networks Incubator Group (SSN-XG) [7] is also working on developing an ontology for describing sensors. Effective description of sensors, observations, and measurement data, and utilizing semantic web technologies for this purpose, are fundamental steps to construct semantic sensor networks. However, associating this data to the existing concepts on the web and reasoning the data is also an important task to make this information widely available for different applications, front-end services, and data consumers.

Linked sensor middleware (LSM) [9] provides many functionalities, such as (i) wrappers for real-time data collection and publishing; (ii) a web interface for data annotation and visualization; and (iii) a Simple Protocol and RDF Query Language (SPARQL) endpoint for querying unified linked stream data and linked data. It facilitates the integration of sensed data with data from other sources, both sensor stream sources and data are being enriched with semantic descriptions, creating linked stream data. Sense2Web [18,19] supports flexible and interoperable descriptions and provides associations of different sensor data ontologies to resources described on the semantic web and the web of data, it focuses on publishing linked-data to describe sensors and link them to other existing resources on the web. SPITFIRE [8] is a Semantic Web of Things framework which provides abstractions for things, fundamental services for search and annotation, as well as integrating sensors and things into the LOD cloud using the linked data principles. Moreover, SPITFIRE also provides a semi-automatic creation of semantic sensor descriptions by calculating the similarities and correlations of the sensing patterns between sensors.

Gyrard et al. [20] proposes a cross-domain IoT application development platform—the M3 framework. M3 [21] is based on semantic web technology to explicitly describe the meaning of sensor measurements in a unified way by reusing and combining domain ontologies. The M3 taxonomy describes sensor names, measurement names, units, and IoT applicative domains. This is a kind of dictionary for IoT to easily deal with synonyms, etc. This M3 taxonomy is implemented as an ontology extending the W3C SSN ontology, which is a cornerstone component to semantically annotate data and extract meaningful information from IoT/sensor data. LOV4IoT [22] designs the Linked Open Vocabularies for Internet of Things (LOV4IoT) dataset. Moreover, it also proposes a dataset of interoperable domain rules to deduce high-level abstractions from sensor data by designing the Sensor-based Linked Open Rules (S-LOR). The two components are integrated within the M3 framework.

Ploennigs et al. [23] presents an architecture and approach that illustrates how semantic sensor networks, semantic web technologies, and reasoning can help in real-world applications to automatically derive complex models for analytics tasks, such as event processing and diagnostics. The work extends the SSN ontology to enable detection and diagnosis of abnormal building behaviors.

### 2.2. Knowledge Extraction and KB Construction on Sensory Data

Some studies have proposed machine learning models and frameworks to extract and annotate knowledge from streaming sensor data. Wang et al. [24] proposes to model time series data using a novel pattern-based hidden Markov model (pHMM), which aims at revealing a global picture of the system dynamics that generates the time series data. Ganz et al. [25] proposes an automated semantic knowledge acquisition from sensor data. The framework uses an extended k-means clustering method and apply a statistic model to extract and link relevant concepts from the raw sensor data and represent them in the form of a topical ontology. Then it uses a rule-based system to label the concepts and make them understandable for the human user or semantic analysis and reasoning tools and software. The CityPulse framework [14] deals with real-time pattern discovery and prediction analysis. It addresses the specific requirements of business services and user applications in smart city environments. It uses cyber-physical and social data and employs big data analytics and intelligent methods to aggregate, interpret, and extract meaningful knowledge and perceptions from large sets of heterogeneous data streams, as well as providing real-time adaptive urban reasoning services. Längkvist et al. [26] has surveyed unsupervised feature learning and deep learning models for time series modeling, which is feasible for high-level pattern recognition and finding states from streaming sensor data. Xu et al. [27] propose an upper-ontology-based approach for automatically generating an IoT ontology. The method proposes an end-to-end framework for ontology construction based on calculating semantic similarity with input schemas with existing IoT ontologies, e.g., SSN.

Some other studies are not directly used for IoT domain knowledge base construction; however, their methods could still be transferred for semantic annotation and extraction on structural metadata of devices. For instance, several EL studies on web tables, web lists, and other relational data are highly related to the automatic annotation task on WoT metadata. Limaye et al. [28] proposed to simultaneously annotate table cells with entities, table columns with types, and pairs of table columns with relations in a knowledge base. They modeled the table annotation problem using a number of interrelated random variables following a suitable joint distribution, and represented them using a probabilistic graphical model. The inference of this task is to search for an assignment of values to variables that maximizes the joint probability, which is NP-hard. They resorted to an approximate algorithm called message-passing [29] to solve this problem. Mulwad et al. [30] also jointly model entity linking, column type identification, and relation extraction using a graphical model, and a semantic message passing algorithm is proposed. TabEL [31] uses a collective classification technique to collectively disambiguate all mentions in a given table. Instead of using a strict mapping of types and relations into a reference knowledge base, TabEL uses soft constraints in its graphical model to sidestep errors introduced by an incomplete or noisy KB.

In conclusion, the state of the art works have established a concrete foundations for our research: the top-down knowledge engineering methods adopt domain ontological knowledge to annotate the representation of WoT resources, and existing ontologies could be partly reused as a reference for constructing a domain knowledge base of most of CPS applications, but extended concepts and relations are still needed for resource composition and causal deduction scenarios, such as anomaly diagnosis and automatic control, while the bottom-up machine learning methods focus on extracting knowledge from existing sensory data or linking descriptive metadata of devices to background KBs. These ideas could be references to enrich and further unify the domain knowledge from existing data with prior knowledge. Thus, this paper will propose a SWoT4CPS framework with knowledge extraction and KB construction modules. The modules will design an ontology by reusing and extending existing ontologies and use the EL-based method to enrich and align domain knowledge to common sense KBs. Moreover, a rule-based reasoning model will be proposed to support WoT resource composition and causal deduction applications in CPS scenarios based on the constructed KB.

## 3. Problem Statement and Requirements

CPS has been vastly utilized in building automation, industry 4.0, or smart city application domains, and IoT devices have been interconnected and need to interact with each other to provide automatic and intelligent services in these scenarios. To achieve better interoperability and smarter decision-making process, semantic IoT domain knowledge are modeled and utilized to represent attributes and raw data of IoT devices, as well as relationships between each other under certain circumstances. Thus, a Semantic Web of Things framework needs to be designed to provide knowledge base construction and reasoning tools for these automatic and intelligent CPS applications. Specifically, to achieve the ultimate goal, there are some challenges to be dealt with:

- SSN is a well-known uniform upper ontology to model a semantic sensor network, and it mainly describes the physical attributes, deployment, and sensing-observing process of sensors. However, CPS scenarios are usually complex and dynamic. For example, in building automation or industry 4.0 applications, it is common that sensors and actuators collaborate with each other in the loop to provide anomaly diagnosis and automatic controlling services, while SSN, or other extended solutions, studied in Section 2 could not cover such use cases particularly well for the reason that monitor-control relationships between devices or cause-effect relationships between anomaly events and observed causes have not been fully considered yet.
- Though the semantic representation of devices could be formatted with domain ontologies, it only specifies the conceptual type that the facts belong to. Since the understandings of the concepts are variable for people who model the services with different backgrounds, the meanings of the facts filled with textual contents are sometimes similar or ambiguous, which needs alignment or disambiguation. For instance, precipitation and rainfall sensors have the same meaning for sensor types, and Peking is another representation of Beijing for a city name. While a common sense KB, such as DBpedia [32], Yago [33], and Freebase [34], have defined such common sense concepts, entities, and relationships, therefore linking domain facts and concepts with common sense KBs could facilitate enlargement and alignment of a domain knowledge base. Moreover, it is more interoperable and unified for human-machine interaction and semantic reasoning with linked common knowledge in domain CPS applications.

Accordingly, our research goals and requirements could be summarized as: firstly, we need to model a uniform semantic WoT ontology for CPS applications by extending SSN and reuse some existing ontologies for interoperability. The ontology needs to describe sensing-observing processes, monitoring-controlling and cause-effect relationships among devices, as well as the physical attributes of sensors and actuators. The knowledge could facilitate the reasoning and decision-making in intelligent services, such as automatic controlling and anomaly diagnosis.

Secondly, we need to provide a (semi-) automatic semantic extraction, linking, and alignment model to interlink domain knowledge with common sense KBs. Specifically, it should annotate the facts of ontological concept/types to the entities in the common sense KB and associate/align ontological concepts/types to the concepts/types in the common sense KB. Moreover, it should also annotate pairs of ontological concepts/types with a binary relation in the common sense KB if the relations exist. If two keys are not involved in any binary relation in our KB, it should determine that as well.

Thirdly, we need to design and implement a demonstration system to assist in building an IoT knowledge base with both domain and linked common knowledge and, to testify to the feasibility and of the platform, it is necessary to implement a semantic rule-based reasoning engine based on the constructed knowledge base to perform anomaly diagnosis and automatic controlling services for a specific CPS application.

## 4. Semantic Web of Things Framework

This section mainly introduces the overall Semantic Web of Things for CPS (SWoT4CPS) framework with proposed hybrid KB construction modules: (1) SWoT-O ontology model and (2) the entity linking model with iterative message passing algorithms used for disambiguating entities and aligning concepts. The semantic search and reasoning models are also proposed based on the constructed hybrid KB.

### 4.1. SWoT4CPS Architecture

**Figure 1:**
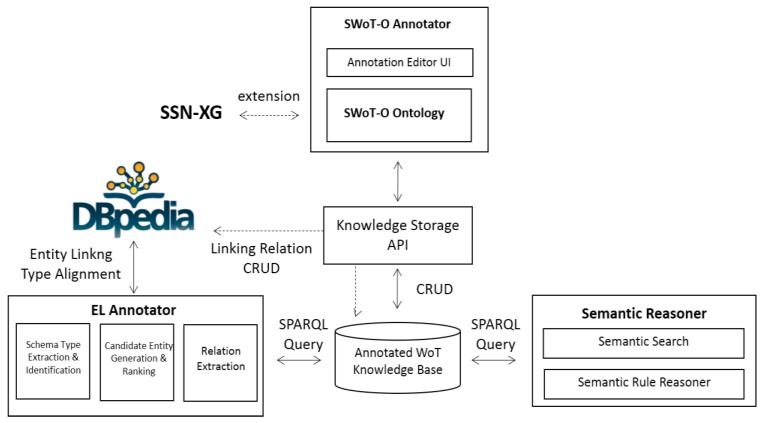
The SWoT system overview.

An overview of the SWoT4CPS framework is depicted in Figure 1. The whole system is composed of four main components: SWoT-O Annotator, EL Annotator, Knowledge Storage, and Semantic Reasoner.

- **SWoT-O Annotator:** This building block is designed for generating metadata representation templates and annotating semantics of WoT resources with the SWoT-O vocabulary. The SWoT-O is extended from the SSN upper ontology and reuses some other IoT ontologies. It describes a sensor-observation-event-rule-actuator-action model that is useful for further reasoning tasks in most intelligent and automatic CPS applications, and it also describes some common necessary attributes of physical devices (including sensors and actuators), such as Location, Ownership, Unit, and DeviceType. The annotator provides a graphical user interface (UI) for the service modeler to create domain services.
- **EL Annotator:** This building block is designed for extracting semantic entities from the metadata of WoT resources and aligning SWoT-O ontologies with the concepts in the common sense KB. In our testbed, DBpedia is used for the referent common sense KB. The input of this module are the annotated WoT instances according to the SWoT-O ontology, and the output are the annotated WoT metadata data with linked entities and aligned ontological concepts to DBpedia. The subtasks are divided into schema type extraction and identification, candidate entity generation and ranking, and relation extraction. The extraction model is extended from the EL [35] framework that is usually used for semantic extraction and alignment on relational Web data.
- **Knowledge Storage:** This building block provides common APIs for storing the knowledge graph into a persistent database. For storage efficiency and scalability, the graph database is proposed to be used. Concepts, properties, entities and relationships in resource description framework (RDF) formats are transferred to graphical data structures. For compatibility with the existing semantic reasoner, RDF-based knowledge storage is also used. The query of the KB is based on a SPARQL-compatible language which could also be used for the graph database.
- **Semantic Reasoner:** This building block is aimed at providing a semantic reasoning capability based on the linked hybrid knowledge. The reasoning process could be based both on logical rules or statistical methods or hybrid methods. The query is based on a SPARQL language, and a list of ranked entities that matches the query are returned. The rule is modeled based on Jena’s rule language, and the reasoning process is based on Jena API and Jena Reasoner [36].

### 4.2. SWoT-O Model for CPS

**Figure 2:**
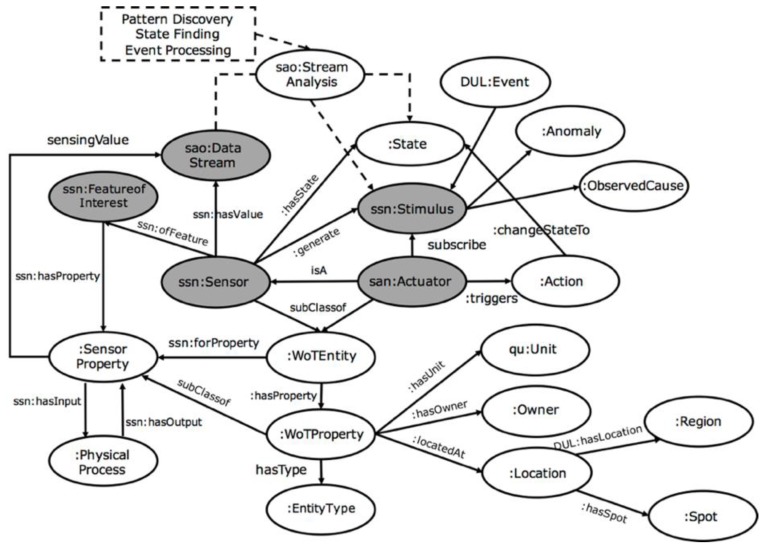
A reference SWoT-O ontology model by extending SSN and reusing other IoT ontologies. Default SWoT namespace have been omitted.

According to the requirements mentioned in Section 2, some key relational triples should be annotated to describe the meta-information of WoT resources. To provide a uniform ontology of CPS applications, the SWoT-based ontology (SWoT-O) [37] (seen in Figure 2) is mainly referred to and extended from SSN ontology, as well as reusing other IoT ontologies, such as a semantic actuator network (SAN) [38] for actuators, stream annotation ontology (SAO) [39] for streaming data, and QUDT ontology [40] for units of measure. The main structure can be categorized as:

- **Sensor-Observation-State-Event:** Sensors observe some objects with *SensorProperty*, which has low-level raw data, and high-level states could be extracted from these observed raw data. The observed system runs and switches among certain states, and when the internal state of the observed system is changed, an event will be generated and published. The high-level state could be extracted by pattern discovery and prediction methods based on streaming mining algorithms, such as unsupervised cluster models [41], hidden Markov models (HMM) [24], or deep learning models [42] with temporal feature representations. In Figure 2, the processing of raw streaming data into high-level states and events are represented with dotted lines, and the line does not represent the exact predicts/relations between the entities but only describes reference methods of how data streams could be transformed into states or events. The SAO ontology is reused to annotate streaming sensory data for further high-level state mining and reasoning with other prior knowledge as [25] proposed.
- **Event-Rule-Actuator-Action:** Since events were generated from sensory observations, the rule will be defined by service providers/developers which describes which event to subscribe to and what action should be triggered by actuators in some condition. The rule is defined for further semantic reasoning by combining forward knowledge with events (*ssn:Sensors :hasState :State* and *:generates ssn:Stimulus*) and backward knowledge with actions (*san:Actuator :triggers :Action*). In an automatic controlling CPS application, the action could change the current state of system to another one. To better describe the actions performed by actuators, the SAN ontology is reused to annotate actuators or controllers. IoT-O [43] could be a reference reusable ontology as well. Since there is no significant difference of using SAN or IoT-O for the actuator concept in our use case, SAN is mainly considered as a reference to reveal the concepts and relations.
- **WoTProperty:** *WoTProperty* describes some basic properties of WoT resources, including *Location*, *EntityType*, *Owner*, and *Unit*, and *SensorProperty* is inherited from *WoTProperty*. *WoTProperty* contains more common sense knowledge and facts, which could be linked to entities and concepts in existing global KBs. In this paper, DBpedia is used as the background KB.
- **FeatureofInterests**: *Feature of Interest* (*FoI*) defines the CPS scenario which is composed of related sensors or actuators. It includes the relations between devices which are interlinked with predefined sets of rules. The rule defines which *WoTProperty* of devices are considered in the scenario and which *SensorProperty* of devices should be activated as the filtering condition of the scenario. In the SWoT4CPS framework, *FoI* will be used as a set of rules to automatically compose related devices as certain CPS scenarios.
- **PhysicalProcess**: The properties of many real world features observed by sensors are related by physical processes. The *Physical Process* models this as a directed relationship of a source *sensorProperty* (*ssn:hasInput*), which influences a target *sensorProperty* (*ssn:hasOutput*) via *itermediateProperty*. It represents the causal relationship between the source property of one device and the target property of another device. For example, in building automation systems, the state of cooling machine or the number of people in the room both influence the indoor energy (*intermediateProperty*), while energy influences the indoor temperature. Hence, by modeling the process chain between devices with their properties and generated events, it could be used for causal reasoning tasks, such as anomaly diagnosis in building automation systems or predictive maintenance for industrial machines.

### 4.3. Semantic Extraction and Alignment for WoT Metadata via Entity Linking

At the SWoT-O Annotator stage, metadata representations of WoT resources are annotated with the SWoT-O vocabulary. Since metadata describes the meta-information about the generated datasets and how they could be accessed and exploited, it is essential to allow discoverability and self-description of sensor datasets. To facilitate WoT resource discovery and composition based on prior knowledge, it is necessary to extend the domain KB of WoT entities with common sense knowledge, such as DBpedia, to improve the results of the semantic-based fuzzy search.

For instance, three devices are annotated by SWoT-O with location information “Beijing”, “Peking”, and “Shanghai”, respectively. Though it explicitly describes where the sensors are located, the relation between “Beijing” and “Shanghai” are missing, as well as the exact type of the location, in the scope of domain knowledge. Thus, it is not applicable to query “all sensors located in China” or “all sensors deployed in the city” if the background knowledge base is not sufficient. While this knowledge exists in DBpedia, and if “Beijing” and “Shanghai” could be linked to the corresponding entities in the DBpedia, the fuzzy discovery of sensors is feasible. Consequently, in the SWoT4CPS framework, the challenges are how to extract similar semantics of domain facts with entities in common KBs, as well as aligning the domain concepts with the entity types in common KBs.

#### 4.3.1. Approach and Model

**Figure 3:**
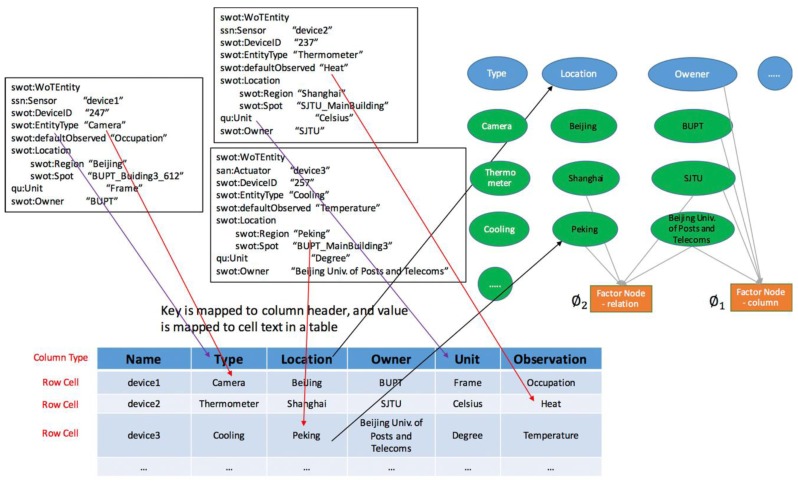
WoT metadata is semi-structural relational data with key-value pairs. The EL Annotator can transfer the WoT metadata into tabular data and perform entity linking tasks. EL tasks use a probabilistic graphical model to jointly inference the linking and mapping.

Some previous works have proposed methods to annotate entities, types, and relations from web tables or relational data [28,31]. Similar to these studies, the ontological WoT metadata with SWoT-O are structured hierarchical data, which can also be modeled as domain semantic web tables with headers and cell values (as shown in Figure 3).

The EL Annotator queries the background KB sources to generate initial ranked lists of candidate assignments for schemas, content values, and relations between schemas. Once candidate assignments are generated, the joint inference component uses a probabilistic graphical model to capture the correlation between schemas, content values, and schema relations to make class, entity, and relation assignments. Details of the model are described as follows:

- Candidate entity generation and ranking

The candidate generation contains *Query and Rank* module which generates a set of candidate assignments for column types, cell texts, and relations between columns from a given KB.

**Figure 4:**
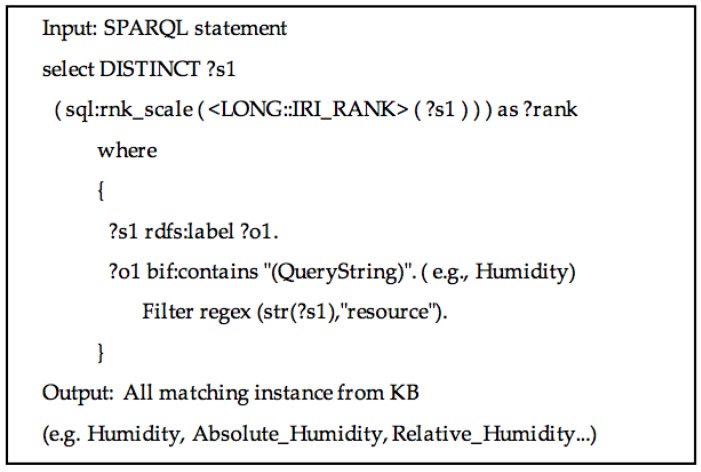
The SPARQL query for generating candidate entities for cell text.

The query module generates an initial ranked list of candidate assignments for the cell values using data from DBpedia. DBpedia could be accessed via its open endpoint through its URL [44]. The SPARQL query is formulated as Figure 4. The cell text is used as SPARQL query inputs defined by DBpedia [45], and the outputs are limited with predefined conditions. The candidate entity should be a “resource” category and the query string should fuzzily match the content of *rdfs:label*. According to the rule, an initial ranked list of entities for each SPARQL query statement are generated, along with the entity popularity score.

The ground truth ranking is expected as high as possible, while the initial ranked list of entities is a disordered one which does not fully meet our requirements. For instance, when we input cell text “*USA*” into the query module as a query string, the entity “*United_States*” ranking first in the return list is expected. However, what we actually get from the top of the return list are “*Democratic_Party_*(*United_states*)”, “*Republican_Party_*(*United_States*)”, etc. The target entity “*United_States*” ranks out of the top 50 of initial ranked list.

To raise the target entity’s ranking, we train an open-sourced support vector machine (SVM) ranking classifier [46] that scores how likely a candidate entity is to be a correct assignment for a given query string, and we use this prepared model to re-rank the initial candidate entities list. The SVM ranking classifier is a pairwise classifier, and it is trained on a set of string similarity and entity popularity metrics as its feature vectors, which we present as follows:

- **Entity Popularity: **Entity popularity *P_pro_* is a kind of prior probability feature. It helps in cases where disambiguation is difficult due to the existence of entities having similar names. It has already been integrated into the DBpedia so that we can access it from the query module directly. The popularity score of an entity is based on how frequently they are referenced by other entities. Entity popularity score is increased as a function of the score of the referencing entity, that is, the higher popularity score an entity obtains, the greater the reference to this entity.
- **String Similarity:** In contrast to popularity, string similarity $Sim_{s}\left(k\right)$ provides a syntactic comparison between cell text and candidate entities. Many candidates do not fully match cell text, so we select five common features such as Keyword Coefficence (KC), Levenshtein Distance (LD), Dice Score (DS), String Length (SL), and Word Contain (WC) to measure string similarities between this pairs. Several 0/1 metric checks are developed to measure whether entities fully contain the words in the cell text, or whether entities are equal to the cell text. We also check whether all words in the cell text are found in the same order in the candidate entity.

A *rankingScore* function is defined based on the feature vectors to represent the relevance degree between the target entity and candidate entities. The *rankingScore* function is a linear function with weight α for the entity popularity feature *P_pro_* and weight $\beta_{k}$ for the other five string similarity features *Sim_s_*(*k*). The weights will be pre-trained in a supervised method with labeled datasets.

(1) $$rankingScore=\alpha \times P_{pro}+\overset{4}{\underset{k=0}{\sum }}\beta_{k}\times Sim_{s}\left(k\right)$$

- Candidate type and relation generation

**Figure 5:**
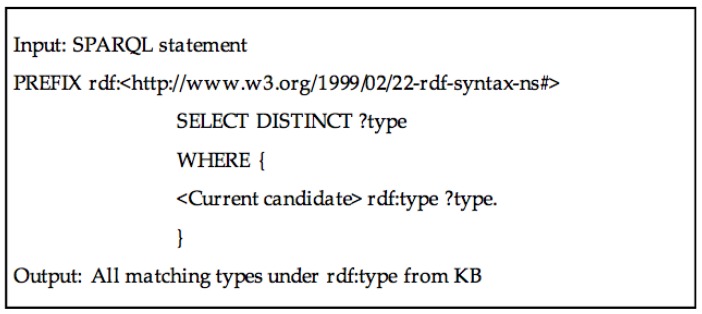
The SPARQL query used for generating candidate types.

For each cell in the input table, we select a candidate entity which ranks at the top of the re-rank list as the current candidate. Then we set another query to the DBpedia endpoint for seeking all types that current candidate belongs to. The SPARQL query for generating candidate types is shown in Figure 5.

**Figure 6:**
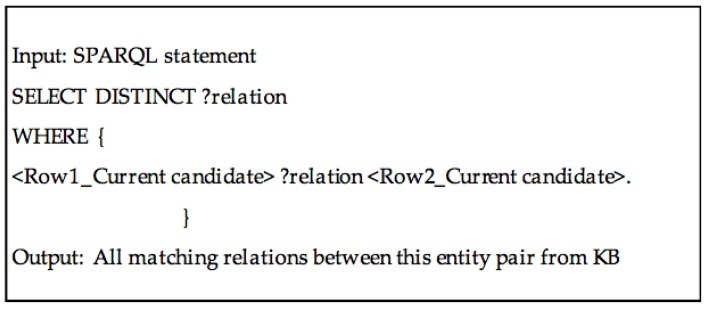
The SPARQL query used for generating candidate relations.

We pairwisely combine input columns and select all combination without repetition. To each column pair combination, we use the links between pairs of current entities to generate candidate relations. For each row in a combination, we obtain each possible relation between the current entities by querying DBpedia in either direction, for example, <*candidateRow1 property1 candidateRow2*> or <*candidateRow2 property2 candidateRow1*>. The candidate relation set for the entire column pair combination is generated by taking a union of candidate relations between individual pairs of row current candidates. The SPARQL query for generating candidate relations is shown in Figure 6.

#### 4.3.2. Joint Inference Model

Once the initial sets of candidate assignments are generated, the joint inference module assigns values to schemas and content values and identifies relations between the schemas. The result is a representation of the meaning of the WoT metadata as a whole. Probabilistic graphical models provide a powerful and convenient framework for expressing a joint probability over a set of variables and performing inference or joint assignment of values to the variables. We represent a set of WoT metadata data with the same domain-specific structures as an undirected Markov network graph in which the schemas and content values represent the variable nodes and the edges between them represent their interactions.

**Figure 7:**
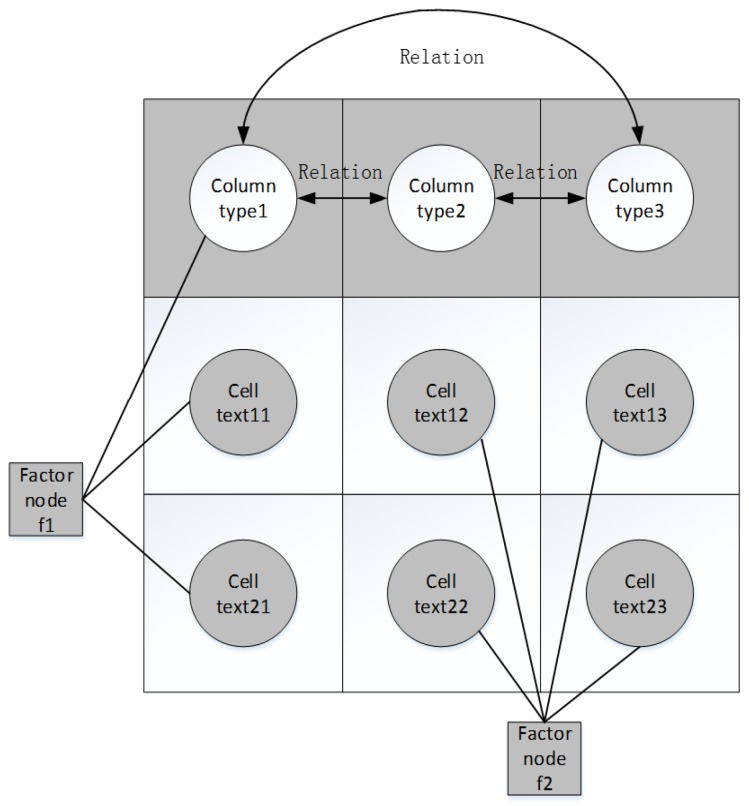
Factor graph of a given relational table.

We propose iterative message passing (IMP) to collectively disambiguate all assignments in a given WoT metadata template. To clearly describe IMP, we first represent the given table as a factor graph which is kind of Probabilistic Graph Model (PGM) that shown in Figure 7. In this factor graph, each solid circle is called cell node and each hollow circle is called column node. Both of them belong to what are known as ‘variable nodes’, and each square node belongs to what is known as a ‘factor node’. All variable nodes in one column can be linked to factor node *f1* and all cell nodes in different two columns can be linked to factor node *f2*. Factor nodes could provide agreement functions on selecting the best assignment to each variable node according to the joint constraints.

IMP is an iterative inference method which reassigns each candidate of a variable node to its maximum-likelihood value. IMP evaluates the likelihood according to the constraints of column type and relation which are relevant to the corresponding variable node. In each iteration, the maximum-likelihood value for each variable node is computed by using its linked factor nodes. Algorithm 1 shows how IMP performs iterative inference over the factor graphical model to find the maximum-likelihood set of referent entities for all assignments. The method initializes each current assigned candidate with an entity ranking at the top of the re-rank list derived from re-rank module (lines 2–4), and then iteratively recomputes constraints between column type, relation, and entity (lines 5–12). Finally, the candidate entity is reassigned until there is no change in assignment to any variable node or the maximum iteration limit is reached (lines 13–15).
**Algorithm 1: Iterative Message Passing**1: Vars ← all variable nodes in graphical model  Factors ← all factor nodes in graphical model 2: **for** all v in Vars **do**
3: e ← currently assigned candidate of v 4: **end for**
5: **for** all f in Factors **do**
6: v’ ← all v in Vars which has a link with f. 7: **if** f==f1 **then**
8: columnConstraint(v’); 9: **else if** f==f2 **then**
10: relationConstraint(v’); 11: **end if**
12: **end for**
13: **for** all v in Vars **do**
14: reassignCandidate(); 15: **end for**
16: Repeat untill all e of Vars no longer change.

The constraints derive from both column and relation. Both constraint functions are defined as factor nodes in our factor model. Since the principles of both column and relation constraints are based on iterative reassignments, we only present Algorithm 2, which describes the column constraint factor, as an example. Algorithm 2 shows how the column constraint factor works, the factor first elects a most common type as the column type annotation through majority voting process (lines 2–13). The query function in line 4 is available in Section 4.3.1. If more than one type obtains the same vote score, the function will obtain their granularity, which is computed by counting the number of instances that belong to the type. The more specific a type is, the fewer instance it obtains. IMP selects the type with maximum vote score and minimum granularity as the column annotation, which means all assignments in this column should have this type. Then, the column constraint is used as feedback. The factor node will send a change message along with the target type to those variable nodes, whose current assigned candidate’s type does not comply with the column annotation.

The relation constraint process is similar to the column constraint. We only list the difference here. Compared to the column constraint, the difference is that relation constraint generates candidate relations with the current assignment of cell nodes in the same row of two columns at first, and then sends a message to both cell nodes. The query is available in Section 4.3.1. The relation can be established in both directions. When a relation annotation between two columns is elected, IMP should take both directions into account.
**Algorithm 2: Column Constraint Function****function columnConstraint(VariableNodeSet v’)**
1: E ← all e of v’//(e is generated in Algorithm 1) 2: Map ← Create a key-value mapping, key represent  type and value represent vote 3: **for** all e in E **do**
4: T ← queryColumnTypes(e); 5: **for** all t in T **do**
6: **if** Map.contains(t) **then**
7: t.value += 1 8: **else**
9: Map.put(t, 1) 10: **end if**
11: **end for**
12: **end for**
13: topVote ← max votes in all t.value 14: T_high_ ← all t where t.value == topVote 15: **for** all t in T_high_
**do**
16: t.granularity ← searchGranularity(t); 17: **end for**
18: top_col ← t with maximum t.value and minimum  t.granularity 19: topScore ← top_col.value/number of rows 20: **if** topScore <= threshold **then**
21: send low-confidence and unchanged messages  to all variable nodes in this column. 22: column annotation ← “no-annotation” 23: **else**
24: column annotation ← top_col 25: **end if**
26: **for** all e in E **do**
27: **if** e.T.contains(top_col) **then**
28: send change message along with top_col as  the type for entity that e should update to 29: **else**
30: send the unchanged message. 31: **end if**
32: **end for**
 **end function**

### 4.4. Semantic WoT Search and Reasoning

To perform semantic sensor search, anomaly diagnosis and automatic control tasks, the Apache Jena reasoner is used for SPARQL query and rule-based semantic reasoning based on the prior knowledge. The details of the process are described as follows:

#### 4.4.1. Semantic Search for WoT Resource Discovery

Since domain knowledge are linked to common sense knowledge via the EL model—for example, the instances of *Region*, *SensorType*, *Owner*, and *Unit* are linked and aligned to entities of *Location*, *Organization*, and *UnitofMeasurement* in DBpedia—the common relationships are inherited to the domain knowledge as well. The enriched knowledge could be used to facilitate searching for semantic entities which has common relationships. To annotate the linking relationship between domain and common sense knowledge, the *linkTo* property is used to represent the linkage.

**Figure 8:**
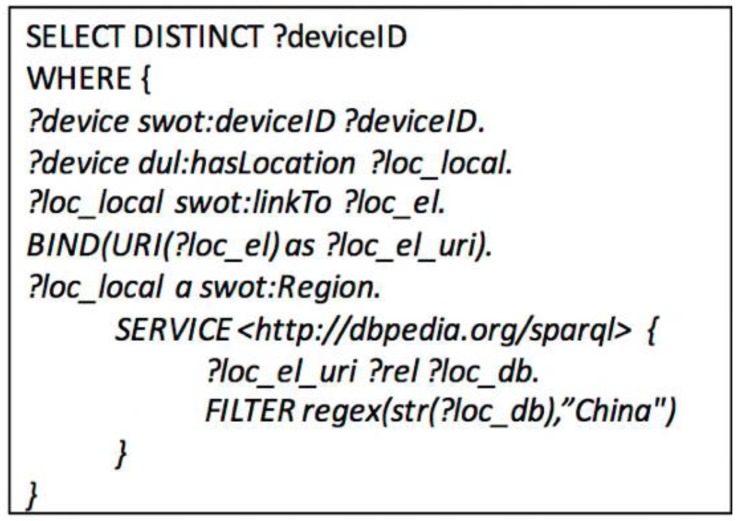
The SPARQL query of a semantic fuzzy search on common sense knowledge.

Searching for WoTEntity instances which have similar properties requires a fuzzy inference according to their potential relationships, while these relationships rely on reasoning according to the common background knowledge. For instance, Beijing and Shanghai are both cities of China, and these knowledge have already been stored in the DBpedia already. If a query of “Searching the sensors located in China”, then the common sense knowledge could be used to inference the correlations among sensors having similar properties. The inference process could be divided into two steps, one which is to search for the domain *Region* instances that has *linkTo* properties with entities in DBpedia, and the other is to search these linked entities that meet the queried relations (located in China). The SPARQL query is illustrated in Figure 8.

#### 4.4.2. Semantic Reasoning for Anomaly Diagnosis and Automatic Control

**Figure 9:**
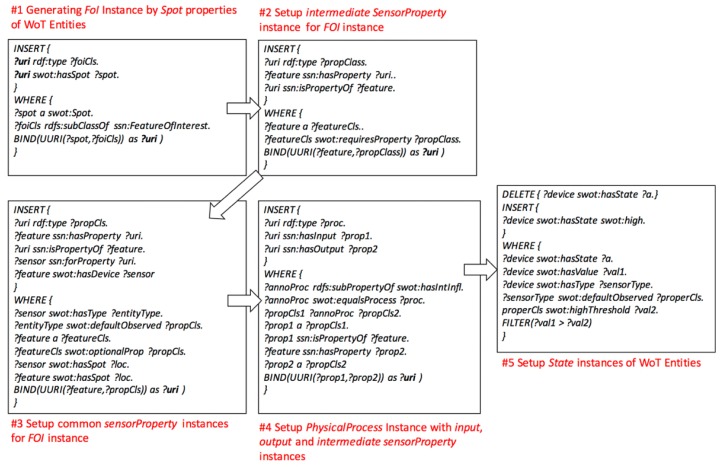
Creating the *FoI* and *PhysicalProcess* model.

**Figure 10:**
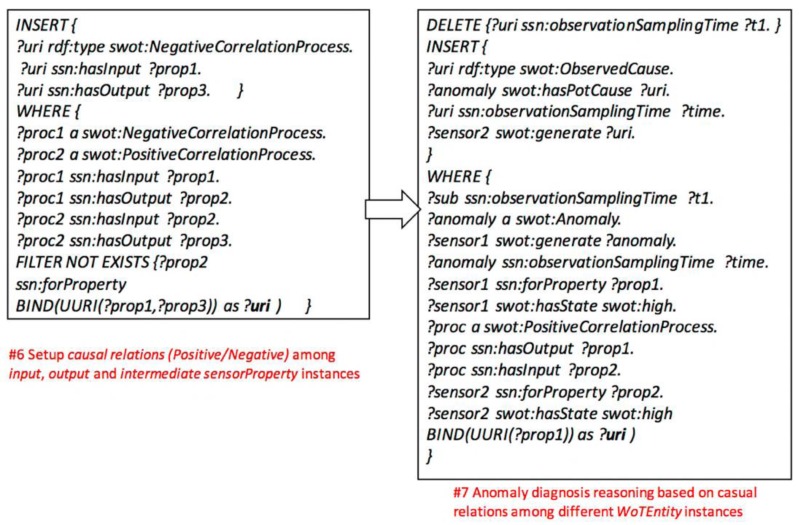
Creating the anomaly diagnosis model.

**Figure 11:**
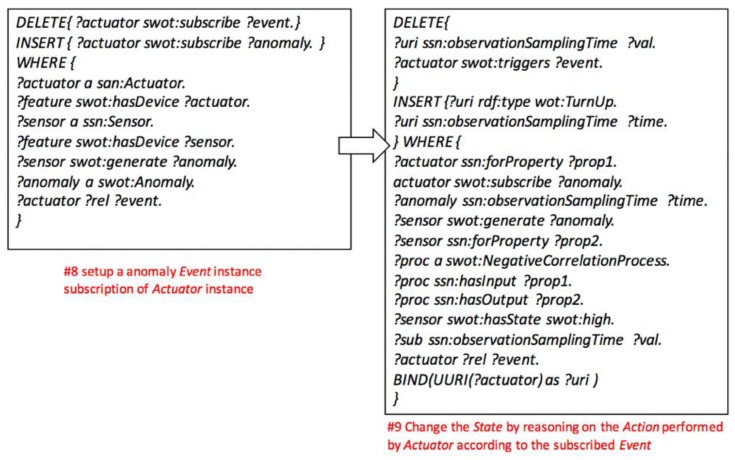
Creating the automatic control model.

After the search and composition of WoT resources, the reasoning will be triggered for anomaly diagnosis and automatic control. The reasoning strategies are based on predefined Jena rules and modeled as SPARUL (SPARQL/Update) [47] statements for execution. The process of the reasoning model could be divided into three parts:

(1) Setup the *FoI* and *PhysicalProcess* models: inferring relationships among *WoTEntity* instances with *sensorProperty* according to configurable rules of *FOI* and *PhysicalProcess* instances. Once the instances of WoTEntity are initialized, the FOI instances will be created, as well as the relations to the *WoTEntity*’s common and intermediate *sensorProperty* instances according to the rule of *FOI* model (#1, #2, and #3 SPARUL codes in Figure 9). To initialize the *PhysicalProcess* instances, the *common* sensorProperty instances are linked to *PhysicalProcess* instances as input and output parameters, while the *intermediate sensorProperty* instances are also linked as intermediate parameters (#4 SPARUL code in Figure 9). Finally, the *State* instances are initialized and linked to *Actuator* instances, and the inferred relations reveal which states the actuator could change (#5 SPARUL code in Figure 9).

(2) Setup anomaly diagnosis model: firstly, inferring causal relationships among input, output, and intermediate *sensorProperty* instances of *WoTEntity* instances. The causal relationships could be modeled as *Positive or Negative* correlations, or more complicated correlations (#6 SPARUL code in Figure 10). Secondly, inference the causes of anomaly effects according to the *PositiveCorrelation* or *NegativeCorrelation* or other correlations among *sensorProperty* instances of corresponding *WoTEntity* instances (#7 SPARUL code in Figure 10).

(3) Setup automatic control model: according to the anomaly diagnosis model, it infers the subscription relationship between *Actuator* instances with anomaly *Event* instances. Once the *Actuator* instance has been initialized, it will subscribe to an anomaly *Event* instance generated by a *Sensor* instance (#8 SPARUL code in Figure 11). Then, according to the *FOI* and *PhysicalProcess* model, it infers the relationships between *Action* instances of *Actuator* instances and *State* instance observed by *Sensor* instances with *sensorProperty* instances (#9 SPARUL code in Figure 11).

## 5. Reference Implementation and Evaluation

### 5.1. Use Case and Proof-of-Concept Implementation

To testify the feasibility and performance, we demonstrate the framework by designing an anomaly diagnosis and temperature automatic control use case for a building automation system. The framework has been open-sourced on Github (https://github.com/minelabwot/SWoT) and more details could be found there as complementary materials.

The scenarios are composed of a temperature sensor, a camera sensor, and a cooling air conditioner (CAC) deployed in each room of buildings at different locations. The temperature sensor can directly detect the indoor temperature, while the CAC can tune the indoor temperature by turning the machine on/off, or the temperature up/down. The camera is used to detect the occupation of the room and the exact number of persons in the room. Our goal is to provide anomaly diagnosis and automatic temperature adjusting services according to indoor temperature anomaly events.

**Figure 12:**
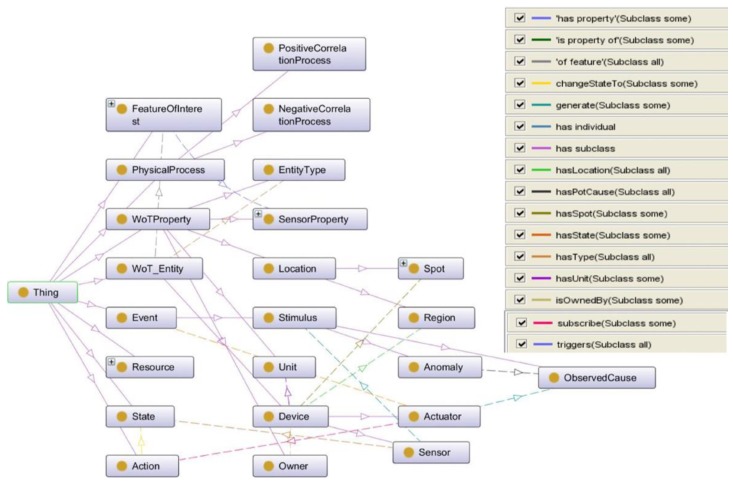
Building up the SWoT-O ontology for a building automation system with Protégé.

**Figure 13:**
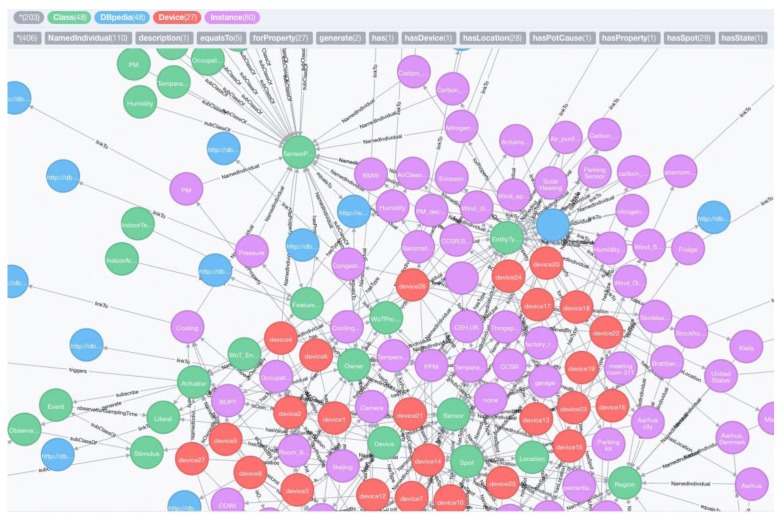
Parts of the EL results stored in Neo4i graph database. The blue circles represent the linked entities and types to DBpedia. It is annotated as the “linkTo” property.

(1) According to SWoT-O vocabulary, we then setup a basic domain knowledge base of how these sensors and actuators collaborate with each other via SWoT-O Annotator (seen in Figure 12). The temperature sensor and camera are annotated as *ssn:Sensor* with *:WoTProperty*, such as *qu:Unit*, *:Location* (*:Region and :Spot*), *:Owner* and *:EntityType*, while the CAC is annotated as *san:Actuator* with *:Action*. The *ssn:FeatureofInterest* is modeled as the target scenario composed with *:SensorProperty* of the temperature sensor, camera, and CAC. The *:PhysicalProcess* is modeled as the causal relation among these devices with their *:SensorProperty* as input and output parameters. In this use case, the causal relations are categorized into two types (:*PositiveCorrelationProcess* and :*NegativeCorrelationProcess*) as a reference knowledge for diagnosing the cause of the anomaly. As a proof-of-concept implementation, Protégé is used as the modeling tool (seen in Figure 12) for SWoT-O ontology, and we implement the SWoT-O Annotator based on J2EE. Neo4j [48] and Jena TDB [49] are used for knowledge graph and RDF triples storage. To validate the SWoT-O, the ontology has been submitted to Linked Open Vocabulary (LOV) [50] and visualized with WebVOWL [51] for linking vocabularies and following the best ontological practices.

(2) To link the knowledge base with common knowledge in DBpedia, we run EL Annotator to execute EL task. In this use case, the instances of *:Region*, *qu:Unit*, *:Onwer* and *:EntityType* are linked to the DBpedia., and the linking relations are stored into both Neo4j and Jena TDB. Figure 13 presents parts of the EL results stored in Neo4j graph database. The data sources of the constructed hybrid KB based on the use case have been published at https://github.com/minelabwot/SWoT.

(3) Based on the hybrid knowledge base, the system will perform semantic search and reasoning tasks. Since devices are created and deployed in a distributed manner, the first step is to query the target devices which could be composed into temperature adjusting and anomaly diagnosis scenario. The second step is to inference the cause of the anomaly once an anomaly event occurs, and the system will automatically adjust the indoor temperature by controlling the CAC. In our case, it is assumed that the anomaly events have been detected via algorithms [24,42,52] and annotated with SWoT-O ontology. According to the model in Section 4.4, an Apache Jena reasoner is used in the implementations. By triggering a “*High_Temperature_Anomaly_Event*”, the reasoner will infer that the *Occupation* of the room and the *State* of the CAC have either positive or negative correlations with the high temperature *Event*, thus, to adjust the temperature to a normal state, the “turn-down” operation of the CAC will be actioned automatically. The demo results can be found at https://github.com/-minelabwot/SWoT/.

### 5.2. Entity Linking Evaluation

**Figure 14:**
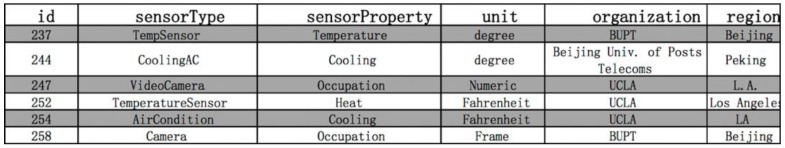
A snapshot of the AG table containing devices with five columns and corresponding row cell texts.

We use *fixedWebTable* from Bhagavatula et al. [31] and WoT metadata generated from our use case as the experimental dataset. The *fixedWebTable* contains 9177 original texts with their annotations from 428 tables extracted from the web. Text data (7500 samples) will be used for pre-training re-ranking model, and the WoT metadata are generated by using the SWoT-O ontolgy in our application denoted as the *Application Generated* (*AG*) *table*. The *AG table* contains 40 devices with five columns and 200 cell texts. The snapshot of the *AG table* is illustrated in Figure 14.

To pre-train the candidate’s re-ranking module, we select 7500 origin texts from *fixedWebTable* as training sets and another 1000 texts of *fixedWebTable* with 100 texts from the *AG Table* as validation sets for tuning the model. The rest of the 677 *fixedWebTable* texts and 100 *AG table* texts are used as test sets. To label the training and validation datasets, the cell texts of the tables are used as input queries to the DBpedia endpoint, and the returned results which fully match the entities are labeled with a high ranking score (five points), and other results are labeled with low score (one point). To preprocess the test datasets, we manually annotate each cell text with ground truth in DBpedia. Then we drop the texts which have no ground truth in DBpedia from our dataset and denote this new test dataset as *D*. Accuracy is chosen as the evaluation metric to score the annotation results, which is standard in information retrieval.

**Figure 15:**
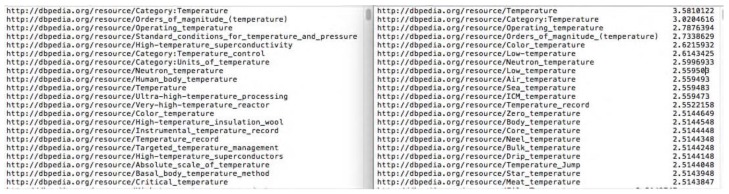
The top-N ranking lists of parts of the candidate entities before and after re-ranking. The left figure is the initial ranking list before re-ranking, while the right figure is the re-ranked top-N lists with ranking scores.

Re-rank is an important step in EL Annotator. It is known that only one assignment matches the ground truth in DBpedia. For each text in *D*, we find its re-ranked candidate list, and evaluate re-rank accuracy by judging whether the top N re-ranked entities contain the ground truth. Figure 15 presents the top-N ranking lists of parts of the candidate entities before and after re-ranking. Table 1 shows the accuracy comparison between two test dataset’s parts in the re-rank module.

To compare with the accuracy that a text’s ground truth ranks in the top N after the re-ranking model, we use the origin rank list which is obtained from querying the DBpedia SPARQL endpoint. The judgment is the same as what we do to the re-ranked candidate list. It can be seen in Table 1, without a candidate entities re-ranking, that the accuracy of “ground truth rank in top 1” has a significant decrease, which means the iterative inference model will take more iterations to converge. For each cell text, we only set the top 10 entities in re-ranked list as the input candidate entities for the iterative inference module. The decreasing accuracy of “ground truth rank in top 10” will directly result in annotation errors due to the absence of the target assignment.

After convergence iterations, every cell text in an input table is assigned to an entity in KB or to a string “no-annotation”. The IMP algorithm could not annotate a nonexistent entity to a text. Thus, we have dropped the entities that are missing ground truth in DBpedia. Then we compare the entity links generated by our system to the ground truth manually annotated before. If the assignment generated from the iterative inference model comply with the ground truth, we consider it as a correct prediction. Otherwise, it is wrong. We present the results regarding accuracy of our algorithm in Table 2, and also present accuracy without the re-rank module in contrast to show how important re-ranking is.

As the input table has several columns, we evaluate entity linking accuracy for each column and find that entity annotations under the column which represents the unit has an extraordinarily low accuracy, only 26.1%, far below the other columns whose mean accuracy is 88%. Lower accuracy is likely due to the lack of relevant data in DBpedia. Although our system might have discovered the correct assignments for column type and relation, if this entity does not have the same type and relation information in DBpedia, the system will miss this correct assignment.

Performance is also evaluated to test how quickly the IMP converges. The number of variable nodes that need to be reassigned decrease to zero after the first two iterations. Two reasons can be used to explain this phenomenon. One is that the re-rank model has such a high accuracy that it can precisely re-rank the target assignment at the top of the re-rank list; the other is that few relations exist between the columns in our test dataset. The number of relations is positively related to the messages that a variable node received. A variable node with several relations may result in an increase in iterations. The average time consumed in each annotation on the WoT table is 4.1 s, and there is a considerable variation depending on the number of rows and columns.

### 5.3. Discussion

We have demonstrated an anomaly diagnosis and automatic temperature control application to testify to the feasibility and performance of the SWoT4CPS framework. Advantages and disadvantages can be concluded as follows:

(1) Compared with research in the state of the art, SWoT-O ontology describes not only the physical attributes, deployment, and sensing/observing process of sensors, but also defines actuators with its actions, as well as monitor-control relationships or cause-effect relationships between them. Based on the prior knowledge, assisted decision-making services could be processed via semantic reasoning, such as anomaly detection and diagnosis, according to the sensing results and with automatically controlling actuators for anomaly discovery. These intelligent services are necessary in CPS applications for building automation, industry 4.0, and smart city scenarios. However, current SWoT-O still needs to be improved by reusing external ontologies. Regarding actuators with their actions for control loops, SAN and IoT-O ontologies could be referred to, while we are not considering very detailed properties for actuators and how to align these concepts or relationships (i.e., *san:actsOn*, *san:actuatorInput*, *san:actuatorOutput*, and *iot-o:Operation*) to current SWoT-O ontologies. It could be a future work to complement the SWoT-O, and its usage in automatic control/service composition applications should be modeled and designed when details of actions and services need to be planned and invoked.

(2) The EL method could semi-automatically link domain knowledge to common sense knowledge, which enlarges and improves the interoperability of the domain KB. Based on the hybrid knowledge base, CPS applications could perform a two-stage reasoning task with both common sense knowledge reasoning for semantic relatedness and domain knowledge reasoning for causal relations. According to the experiment, the EL method has more than 90% accuracy and the time complexity is at a tolerant level for offline annotation tasks. The results show that the framework could improve the effectiveness and efficiency of semantic annotation and KB construction for CPS applications.

(3) Though the EL method provides a semi-automatic and scalable model, existing prior knowledge for sensor networks or CPS applications are not sufficient enough, currently, to adopt machine learning methods to train the model for extracting semantic entities from WoT representations, or linking entities and aligning concepts with the global KB. Consequently, the current EL model could only extract some of the entities with types from the WoT metadata, while the relation extraction among these entities or types does not work well. Thus, a hybrid framework with both manual knowledge engineering methods and semi-automatic learning methods are more practical for a bootstrap for constructing a uniform KB for CPS applications.

(4) Compared with current SWoT4CPS framework with static annotated knowledge, semantics have not been fully exploited from streaming data yet. Section 2 listed the state of the art works on this issue, and these studies mainly use ontology to annotate directly on the raw sensory data and use extended SPARQL (e.g., CQELS [53], C-SPARQL [54], EP-SPARQL [55], etc.) to efficiently query the RDF stream for semantic event processing. It focuses on mining and reasoning on online streaming data in a relatively short time window for real-time event-driven applications, while historical time series data reflect dynamic states and the state transfer of the observed system. Thus, mining semantics from multivariate time series could enrich the CPS KB with more dynamic knowledge, which could reveal how the system works. These knowledge and predicting models will be very useful for predicting future states of the system, finding correlations between observed properties, and making decision for further automatic operations. Thus, integrating event processing models with time series mining models will be a future work for online semantics extraction, annotation, and prediction of sensory data.

## 6. Conclusions

This paper proposes the SWoT4CPS framework, and it provides a hybrid solution with both ontological engineering methods by extending SSN and machine learning methods based on an entity linking (EL) model. To provide a uniform ontological representation of WoT resources in CPS applications, SWoT-O ontology is modeled for further semantic logical reasoning. To link and align the domain knowledge with global KB, an EL framework for WoT metadata is proposed based on a probabilistic graph model. The inference of the model is based on a message-passing algorithm to calculate the joint probability of contexts inside the representation of WoT metadata. To testify to the feasibility and performance, we demonstrate the framework by implementing a temperature automatic control and anomaly diagnosis use case in a building automation system based on the SWoT-O ontology. Evaluation results on the EL method show that linking domain knowledge to DBpedia has a relative high accuracy and the time complexity is at a tolerant level.

Future work of our framework will focus on following existing best practices and methodologies of state of art’s work to improve the SWoT-O ontology by better reusing external ontologies, especially for actuators with their abilities to deal with automatic control loops. Furthermore, designing and integrating semantic streaming mining models from multivariate time series are also future challenges. Especially, pattern recognition and semantic reasoning methods based on machine learning and deep learning models will be further investigated and researched in the future.

## Figures and Tables

**Table 1:** Accuracies of ground truth ranked in top 10 and top 1 for the SVM ranking model. The values in bold are re-ranked accuracies which show that re-ranking model improves the results compared to initial ranks.

Dataset	Condition	Ground Truth Rank in Top 10	Ground Truth Rank in Top 1
fixedWebTable	initial rank	90.3%	57.9%
	re-ranked	**94.7%**	**71.8%**
AG Table	initial rank	84.3%	58.4%
	re-ranked	**94.4%**	**83.1%**

**Table 2:** The accuracies of entity annotations on AG Table datasets based on using only IMP model and IMP plus Re-Rank model. The value in bold is the accuracy of IMP plus Re-Rank model which show that the result improves a lot compared with using only IMP model.

Dataset	IMP Only	IMP + Re-Rank
AG Table	57.2%	**74.2%**
